# Nomogram based on homogeneous and heterogeneous associated factors for predicting distant metastases in patients with colorectal cancer

**DOI:** 10.1186/s12957-021-02140-6

**Published:** 2021-01-27

**Authors:** Tianwen Luo, Yutong Wang, Xuefeng Shan, Ye Bai, Chun Huang, Guangcan Li, Hongmei Wang

**Affiliations:** 1 Department of Medical and Education Office, The First Affiliated Hospital of Chongqing Medical University, Chongqing, People’s Republic of China; 2 Department of Epidemiology and Biostatistics, The First Affiliated Hospital, Army Medical University, Chongqing, People’s Republic of China; 3 Department of Pharmacy, The First Affiliated Hospital of Chongqing Medical University, Chongqing, People’s Republic of China; 4 Department of Epidemiology and Health Statistics, School of Public Health and Management, Chongqing Medical University, Chongqing, People’s Republic of China; 5 Department of Cardiothoracic Surgery, The First Affiliated Hospital of Chongqing Medical University, Chongqing, People’s Republic of China; 6 Department of Pharmacy, The People’s Hospital of Kaizhou District, No. 8, Ankang Road, Hanfeng Street, Kaizhou District, Chongqing, 405400 People’s Republic of China; 7 Department of Pharmacy, The First Affiliated Hospital of Chongqing Medical University, No. 1, Youyi Road, Yuanjiagang, Yuzhong District, Chongqing, 400016 People’s Republic of China

**Keywords:** Colorectal cancer, Metastases, Incidence, SEER, Nomogram

## Abstract

**Background:**

The identification of the homogeneous and heterogeneous risk factors for different types of metastases in colorectal cancer (CRC) may shed light on the aetiology and help individualize prophylactic treatment. The present study characterized the incidence differences and identified the homogeneous and heterogeneous risk factors associated with distant metastases in CRC.

**Methods:**

CRC patients registered in the SEER database between 2010 and 2016 were included in this study. Logistic regression was used to analyse homogeneous and heterogeneous risk factors for the occurrence of different types of metastases. Nomograms were constructed to predict the risk for developing metastases, and the performance was quantitatively assessed using the receiver operating characteristics (ROC) curve and calibration curve.

**Results:**

A total of 204,595 eligible CRC patients were included in our study, and 17.07% of them had distant metastases. The overall incidences of liver metastases, lung metastases, bone metastases, and brain metastases were 15.34%, 5.22%, 1.26%, and 0.29%, respectively. The incidence of distant metastases differed by age, gender, and the original CRC sites. Poorly differentiated grade, more lymphatic metastasis, higher carcinoembryonic antigen (CEA), and different metastatic organs were all positively associated with four patterns of metastases. In contrast, age, sex, race, insurance status, position, and T stage were heterogeneously associated with metastases. The calibration and ROC curves exhibited good performance for predicting distant metastases.

**Conclusions:**

The incidence of distant metastases in CRC exhibited distinct differences, and the patients had homogeneous and heterogeneous associated risk factors. Although limited risk factors were included in the present study, the established nomogram showed good prediction performance.

**Supplementary Information:**

The online version contains supplementary material available at 10.1186/s12957-021-02140-6.

## Background

Colorectal cancer (CRC) ranks as the third most commonly diagnosed malignancy and the second leading cause of cancer death worldwide [1]. Approximately 1.8 million new cases and 880,000 deaths were estimated by the International Agency for Research on Cancer in 2018 [2]. Distant metastases have a significant impact on the prognosis of CRC. A previous study showed that the 5-year survival rate of patients with distant metastases was only 14%, and the survival rate of patients with localized stage CRC was 90% [3]. Studies investigating the incidence of liver, lung, bone, and brain metastases in CRC are relatively rare, and the findings remain controversial [4–7]. Few studies investigated the risk factors for specific organ metastases in CRC [8, 9]. Overall, there has been no systematic research examining the homogeneous and heterogeneous risk factors for distant metastases in patients with CRC. The predictive models are not ideal due to the limited sample size.

The present study characterized the incidence differences and the differences in risk factors for liver, lung, bone, and brain synchronous distant metastases in CRC patients based on the Surveillance, Epidemiology, and End Results (SEER) database. We constructed a nomogram model to predict the probability of specific organ metastases. The early detection of risk factors for distant metastases may predict the probability of metastases, improve survival, and help obtain a deeper understanding of the pathogenesis of different organ metastases in CRC patients.

## Methods

### Population

Data in this population-based study were retrieved from the US National Cancer Institute (NCI) open public database, the SEER database. Data collection for metastatic sites, such as the liver and lung, started in 2010, and the latest data are available through December 31, 2016. Distant metastases in the SEER database were collected at the initial diagnosis of CRC, which means that the distant metastases were all synchronous metastases. CRC patients who were diagnosed between 2010 and 2016 and patients with liver, lung, bone, and brain synchronous metastases were included in the present study. Cases diagnosed at autopsy or via death certificates, with unspecified follow-up, or unknown first tumour site were excluded. Patients without distant metastasis information were also excluded. Because this study used previously collected data, it was exempt from the ethical review of the ethics board of the First Affiliated Hospital of Chongqing Medical University. SEER*Stat version 8.3.5 (https://seer.cancer.gov/seerstat/) (Information Management Service, Inc. Calverton, MD, USA) was used for case listing.

### Statistical analysis

Quantitative data are presented as the means ± standard deviation (SD), and categorical data are described as numbers and percentages (*N*, %). Univariate and multivariate logistic regression models were used to determine the factors associated with distant metastases in CRC. Factors with *P* < 0.05 were incorporated into the multivariable regression model. Based on the results of the logistic analysis, the intersection of the risk factors for the four types of metastases was used to identify homogenous or heterogeneous factors. Predictive nomograms for liver metastases, lung metastases, bone metastases, and brain metastases were formulated. Receiver operating characteristics (ROC) curve, area under the curve (AUC), C-index, and calibration curves were used to evaluate their performance. Statistically significant levels were two-tailed and set at *p* < 0.05. Statistical analyses were performed using the IBM Statistical Package for the Social Sciences (SPSS) version 23.0 software package for Windows (SPSS, Inc., Chicago, IL, USA). The nomogram was plotted using the “rms” and “dca. R” package in R version 3.4.1 (R Foundation for Statistical Computing, Vienna, Austria; www.r-project.org), and all ROC curves were generated using MedCalc 18.2.1.

## Results

### Demographic and clinical characteristics

**Fig. 1:**
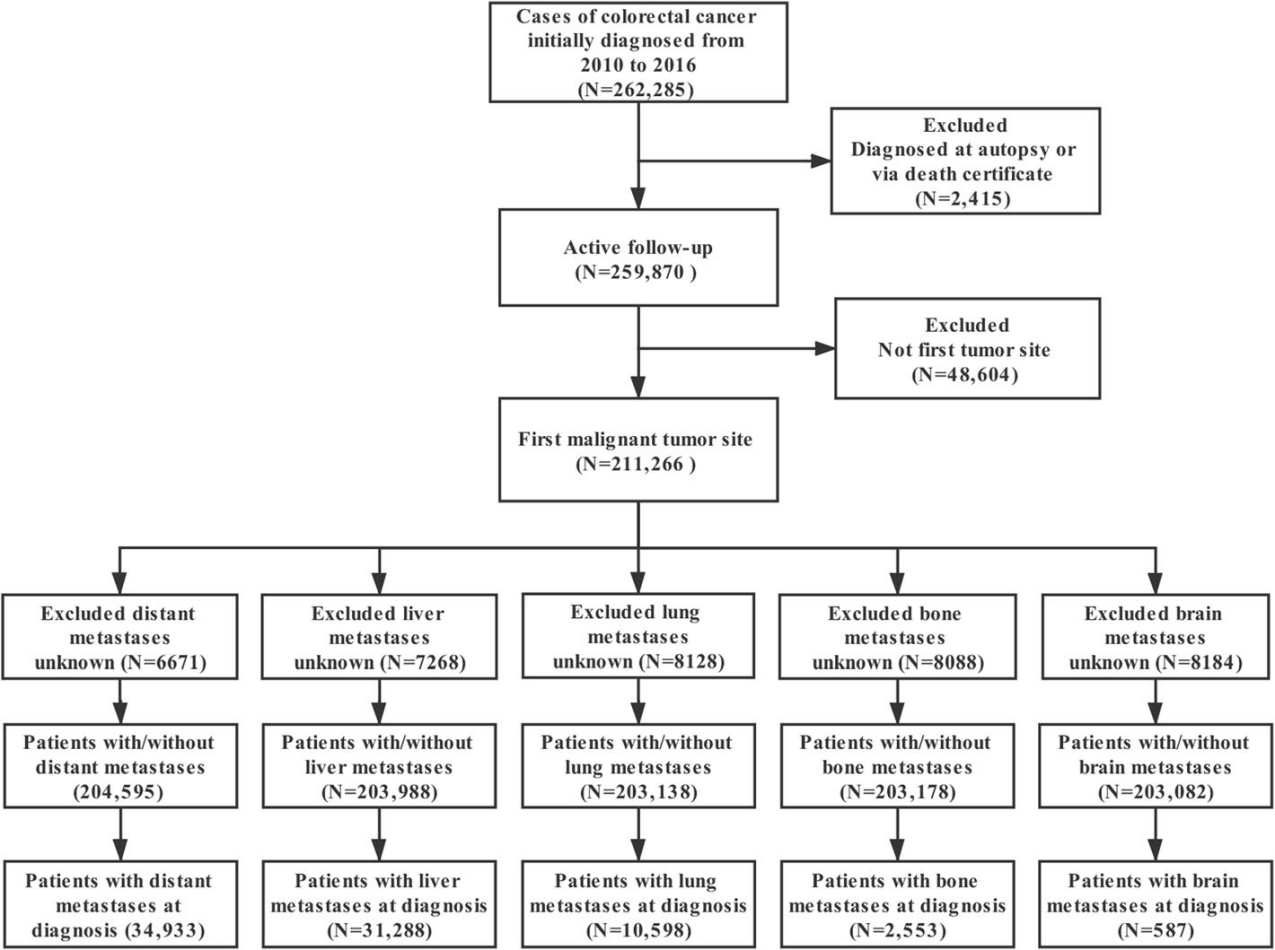
Flowchart of colorectal cancer patient selection

A total of 211,266 eligible CRC patients from 2010 to 2016 were selected from the SEER database. After excluding patients with unknown distant metastasis information, this study ultimately included 204,595 patients with/without distant metastases. Of these patients, 31,288 cases had liver metastases, 10,598 cases had lung metastases, 2553 had bone metastases, and 587 had brain metastases (Fig. 1). The mean age of all patients with distant metastases was 64.92 ± 14.33 years (range 4 to 108), 52.0% of the patients were male (*N* = 106,488), and 48.0% were female (*N* = 98,107). Most of the patients were white (76.8%, *N* = 157,037), and 51.2% were married (*N* = 104,780). Rectal cancer (23.43%, *N* = 47,933) was the most frequent tumour among the CRC patients. Most CRC patients (41.55%, *N* = 85,002) had stage T3 cancer, and 58.89% of patients (*N* = 120,476) had grade IV cancer. The detailed demographic and clinical characteristics are displayed in Table 1.

**Table 1:** Baseline demographic and related clinical characteristics of patients diagnosed with colorectal cancer

Subject characteristics	Total distant metastases (*N* = 204,595)		Liver met (*N* = 203,998)		Lung met (*N* = 203,138)		Bone met (*N* = 203,178)		Brain met (*N* = 203,082)	
	With met (*N* = 34,933, 17.07%)	Without met (*N* = 169,662, 82.93%)	With liver met (*N* = 31,288, 15.34%)	Without liver met (*N* = 172,710, 84.66%)	With lung met (*N* = 10,598, 5.22%)	Without lung met (*N* = 192,540, 94.78%)	With bone met (*N* = 2553, 1.26%)	Without none met (*N* = 200,625, 98.74%)	With brain met (*N* = 587, 0.29%)	Without brain met (*N* = 202,495, 99.71%)
Age (years)										
≤ 50	5936 (18.6)	18506 (81.4)	5391 (16.9)	26432 (83.1)	1587 (5.0)	30130 (95.0)	431 (1.4)	31293 (98.6)	80 (0.3)	31637 (99.7)
51–60	8382 (17.9)	38424 (82.1)	7635 (16.3)	39074 (83.7)	2517 (5.4)	44034 (94.6)	637 (1.4)	45918 (98.6)	161 (0.3)	46361 (99.7)
61–70	9035 (17.3)	43185 (82.7)	8076 (15.5)	44002 (84.5)	2828 (5.4)	49063 (94.6)	698 (1.3)	51197 (98.7)	178 (0.3)	51707 (99.7)
71–80	6460 (15.7)	34737 (84.3)	5702 (13.9)	35363 (86.1)	2088 (5.1)	38790 (94.9)	476 (1.2)	40411 (98.8)	101 (0.2)	40761 (99.8)
81–90	4269 (15.5)	23355 (84.5)	3750 (13.6)	23758 (86.4)	1319 (4.8)	26010 (95.2)	252 (0.9)	27104 (99.1)	59 (0.2)	27275 (99.8)
≥ 91	851 (17.5)	4007 (82.5)	734 (15.2)	4081 (84.8)	259 (5.4)	4513 (94.6)	59 (1.2)	4702 (98.8)	8 (0.2)	4754 (99.8)
Sex										
Female	15660 (16.0)	82447 (84.0)	13895 (14.2)	83937 (85.8)	4855 (5.0)	92556 (95.0)	1022 (1.0)	96411 (99.0)	279 (0.3)	97106 (99.7)
Male	19273 (18.1)	87215 (81.9)	17393 (16.4)	88773 (83.6)	5743 (5.4)	99984 (94.6)	1531 (1.4)	104214 (98.6)	308 (0.3)	105389 (99.7)
Race										
White	26234 (16.7)	130803 (83.3)	23459 (15.0)	133131 (85.0)	7795 (5.0)	148082 (95.0)	1906 (1.2)	154045 (98.8)	474 (0.3)	155391 (99.7)
Black	5400 (21.1)	20177 (78.9)	4917 (19.3)	20580 (80.7)	1702 (6.7)	23721 (93.3)	402 (1.6)	25019 (98.4)	68 (0.3)	25353 (99.7)
Others^a^	3205 (16.1)	16684 (83.9)	2827 (14.3)	16995 (85.7)	1078 (5.5)	18677 (94.5)	239 (1.2)	19484 (98.8)	45 (0.2)	19668 (99.8)
Unknown	94 (4.5)	1998 (95.5)	85 (4.1)	2004 (95.9)	23 (1.1)	2060 (98.9)	6 (0.3)	2077 (99.7)	0 (0.0)	2083 (100.0)
Marital status										
Unmarried^b^	16189 (18.7)	70408 (81.3)	14428 (16.7)	71859 (83.3)	5136 (6.0)	80725 (94.0)	1190 (1.4)	84657 (98.6)	276 (0.3)	85517 (99.7)
Married	16944 (16.2)	87836 (83.8)	15277 (14.6)	89255 (85.4)	4884 (4.7)	99287 (95.3)	1231 (1.2)	102978 (98.8)	282 (0.3)	103888 (99.7)
Unknown	1800 (13.6)	11418 (86.4)	1583 (12.0)	11596 (88.0)	578 (4.4)	12528 (95.6)	132 (1.0)	12990 (99.0)	29 (0.2)	13090 (99.8)
Insurance status										
Insured	27152 (16.0)	142570 (84.0)	24360 (14.4)	144894 (85.6)	8087 (4.8)	160490 (95.2)	1922 (1.1)	166706 (98.9)	452 (0.3)	168103 (99.7)
Uninsured	1736 (25.3)	5127 (74.7)	1558 (22.8)	5281 (77.2)	589 (8.6)	6229 (91.4)	137 (2.0)	6687 (98.0)	104 (0.4)	27597 (99.6)
Any Medic aid	6045 (21.6)	21965 (78.4)	5370 (19.2)	22535 (80.8)	1922 (6.9)	25821 (93.1)	494 (1.8)	27232 (98.2)	31 (0.5)	6795 (99.5)
Site										
Cecum	5203 (17.2)	25033 (82.8)	4811 (16.0)	25336 (84.0)	1341 (4.5)	28714 (95.5)	321 (1.1)	29747 (98.9)	86 (0.3)	29973 (99.7)
Appendix	280 (4.4)	6022 (95.6)	238 (3.8)	6043 (96.2)	48 (0.8)	6232 (99.2)	29 (0.5)	6255 (99.5)	3 (0.0)	6282 (100.0)
Ascending colon	3914 (14.4)	23235 (85.6)	3585 (13.2)	23495 (86.8)	1001 (3.7)	25976 (96.3)	222 (0.8)	26750 (99.2)	73 (0.3)	26897 (99.7)
Hepatic flexure	1051 (17.0)	5144 (83.0)	964 (15.6)	5214 (84.4)	285 (4.6)	5863 (95.4)	67 (1.1)	6082 (98.9)	20 (0.3)	6121 (99.7)
Transverse colon	1837 (14.7)	10691 (85.3)	1687 (13.5)	10811 (86.5)	432 (3.5)	12011 (96.5)	102 (0.8)	12348 (99.2)	27 (0.2)	12425 (99.8)
Splenic flexure	749 (17.9)	3441 (82.1)	701 (16.8)	3476 (83.2)	170 (4.1)	3993 (95.9)	27 (0.6)	4133 (99.4)	4 (0.1)	4160 (99.9)
Descending colon	1374 (16.2)	7127 (83.8)	1256 (14.8)	7229 (85.2)	355 (4.2)	8101 (95.8)	71 (0.8)	8389 (99.2)	18 (0.2)	8444 (99.8)
Sigmoid colon	7212 (18.1)	32646 (81.9)	6625 (16.7)	33135 (83.3)	1999 (5.0)	37616 (95.0)	448 (1.1)	39172 (98.9)	93 (0.2)	39511 (99.8)
Rectosigmoid junction	3135 (20.3)	12289 (79.7)	2804 (18.2)	12586 (81.8)	1044 (6.8)	14287 (93.2)	231 (1.5)	15097 (98.5)	54 (0.4)	15269 (99.6)
Rectum	7098 (14.8)	40835 (85.2)	5905 (12.3)	41921 (87.7)	2898 (6.1)	44762 (93.9)	690 (1.4)	46988 (98.6)	117 (0.2)	47534 (99.8)
Large intestine^c^	3080 (49.1)	3199 (50.9)	2712 (43.9)	3464 (56.1)	1025 (17.1)	4985 (82.9)	345 (5.7)	5664 (94.3)	92 (1.5)	5879 (98.5)
Histological grade										
Grade I	1420 (6.8)	19577 (93.2)	1253 (6.0)	19717 (94.0)	440 (2.1)	20502 (97.9)	88 (0.4)	20856 (99.6)	16 (0.1)	20924 (99.9)
Grade II	16498 (13.7)	103978 (86.3)	14866 (12.4)	105375 (87.6)	4796 (4.0)	115175 (96.0)	824 (0.7)	119122 (99.3)	208 (0.2)	119707 (99.8)
Grade III	5496 (20.4)	21404 (79.6)	4889 (18.2)	21927 (81.8)	1377 (5.2)	25303 (94.8)	497 (1.9)	26209 (98.1)	119 (0.4)	26569 (99.6)
Grade IV	1037 (19.0)	4432 (81.0)	939 (17.2)	4514 (82.8)	224 (4.1)	5206 (95.9)	79 (1.5)	5353 (98.5)	18 (0.3)	5416 (99.7)
Unknown	10482 (34.1)	20271 (65.9)	9341 (30.6)	21177 (69.4)	3761 (12.5)	26354 (87.5)	1065 (3.5)	29085 (96.5)	226 (0.8)	29879 (99.2)
Lymphatic metastasis										
N0	11445 (9.6)	107837 (90.4)	10133 (8.5)	108992 (91.5)	3631 (3.1)	115298 (96.9)	868 (0.7)	118114 (99.3)	194 (0.2)	118769 (99.8)
N1	11055 (22.2)	38747 (77.8)	9945 (20.0)	39748 (80.0)	3435 (6.9)	46066 (93.1)	751 (1.5)	48781 (98.5)	163 (0.3)	49335 (99.7)
N2	7034 (27.8)	18231 (72.2)	6412 (25.5)	18759 (74.5)	1609 (6.4)	23474 (93.6)	376 (1.5)	24715 (98.5)	90 (0.4)	24988 (99.6)
Unknown	5399 (52.7)	4847 (47.3)	4798 (47.9)	5211 (52.1)	1923 (20.0)	7702 (80.0)	558 (5.8)	9015 (94.2)	140 (1.5)	9403 (98.5)
T stage										
T1	3267 (8.7)	34237 (91.3)	2913 (7.8)	34547 (92.2)	1121 (3.0)	36266 (97.0)	281 (0.8)	37113 (99.2)	57 (0.2)	37333 (99.8)
T2	735 (3.2)	21981 (96.8)	644 (2.8)	22055 (97.2)	175 (0.8)	22522 (99.2)	49 (0.2)	22651 (99.8)	20 (0.1)	22680 (99.9)
T3	10612 (12.5)	74390 (87.5)	9512 (11.2)	75366 (88.8)	2627 (3.1)	82120 (96.9)	457 (0.5)	84308 (99.5)	133 (0.2)	84605 (99.8)
T4	7962 (25.7)	23077 (74.3)	7103 (23.0)	23806 (77.0)	2218 (7.2)	28548 (92.8)	527 (1.7)	30266 (98.3)	102 (0.3)	30675 (99.7)
Unknown	12357 (43.6)	15977 (56.4)	11116 (39.6)	16936 (60.4)	4457 (16.2)	23084 (83.8)	1239 (4.5)	26287 (95.5)	275 (1.0)	27202 (99.0)
CEA										
Negative	3499 (6.1)	54327 (93.9)	2892 (5.0)	54845 (95.0)	899 (1.6)	56789 (98.4)	209 (0.4)	57497 (99.6)	66 (0.1)	57629 (99.9)
Positive	20199 (37.1)	34246 (62.9)	18426 (34.0)	35810 (66.0)	6325 (11.8)	47487 (88.2)	1488 (2.8)	52382 (97.2)	310 (0.6)	53497 (99.4)
Unknown	11235 (12.2)	81089 (87.8)	9970 (10.8)	82055 (89.2)	3374 (3.7)	88264 (96.3)	856 (0.9)	90746 (99.1)	211 (0.2)	91369 (99.8)

*Met* metastases, *CEA* carcinoembryonic antigen, *NA* not available

^a^Includes American Indian/Alaska Native and Asian or Pacific Islander

^b^Includes single, separated, widowed, and divorced

^c^Sites were unclear

### Incidence of synchronous distant metastases

A total of 17.07% (34,933/204,595) of the CRC patients had distant metastases, and the overall incidences of liver metastases, lung metastases, bone metastases, and brain metastases were 15.34% (31,288/203,988), 5.22% (10,598/203,138), 1.26% (2553/203,178), and 0.29% (587/203,082), respectively (*χ*^2^ = 55324.97; *P* < 0.001).

**Fig. 2:**
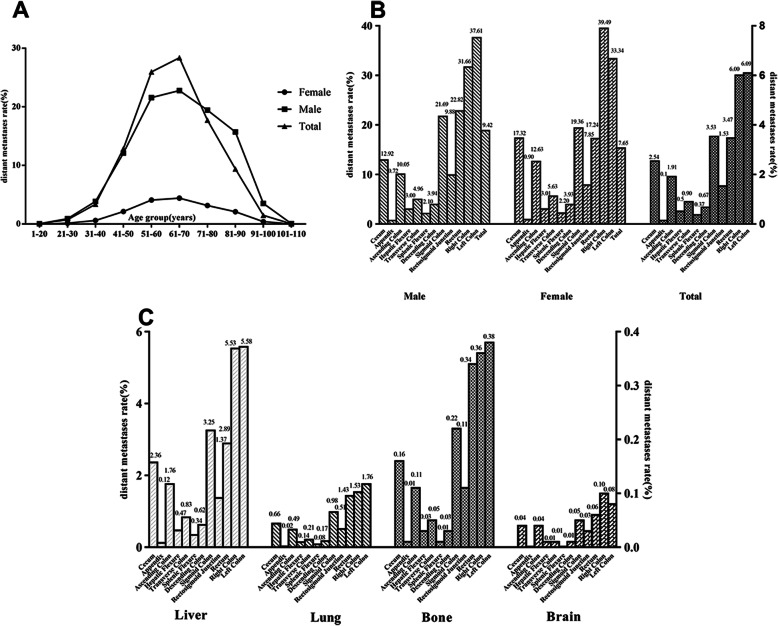
The trend and distribution of distant metastases in colorectal cancer patients. Stratified by age (**a**), different distant metastases by sex (**b**), and different colorectal cancer sites (**c**)

The incidence of distant metastases fluctuated with age. It first increased with the age of patients, and this rising trend was most rapid in patients aged 31 to 51 years. The incidence decreased rapidly in patients > 70 years. The trends of distant metastases in males and females were roughly similar, but the incidence in males aged > 71 years decreased much faster than females (Fig. 2a). Males also had a significantly higher incidence of distant metastases than females (9.42% vs. 7.65%; *P* < 0.001).

The incidence of distant metastases was different by gender and original CRC sites (Fig. 2b). The highest incidence of distant metastases was observed for the sigmoid colon (3.53%), followed by the rectum (3.47%) and cecum (2.54%), and the lowest incidence of distant metastases was from the appendix (0.14%). A similar incidence was observed for the right and left colon sites (6.09% vs. 6.00%, *P* > 0.05). The incidence of total distant metastases in males was higher than females (9.42% vs. 7.65%, *P* < 0.001). However, the incidence of right colon cancer was higher than the left colon cancer in females (39.49% vs. 33.34%, *P* < 0.001), and the opposite incidence was true in males (31.66% vs. 37.61%, *P* < 0.001). The incidence of rectal tumours was lower than the colon, and there was no difference by sex.

For the different colorectal cancer and metastatic sites, the liver was the most common metastatic site for sigmoid colon cancer (3.25%), and lung, bone, and brain were the most common metastatic sites for rectal cancer (1.43%, 0.34%, and 0.06%, respectively). Bone metastases were least frequently observed in splenic flexure cancer (0.01%). The other three organ metastases were the least frequently observed for appendix colon cancer. The left colon had a higher metastatic rate than the right colon, specifically to the liver (5.58% vs 5.53%), lung (1.76% vs 1.53%) and bone (0.38% vs 0.36%). The right colon had a higher incidence rate of brain metastases than the left colon (0.10% vs 0.08%). Overall, the incidence of distant metastases was highest for liver metastases, followed by lung, bone, and brain metastases, but it varied in different original sites (Fig. 2c).

### Risk factors associated with synchronous distant metastatic CRC

**Fig. 3:**
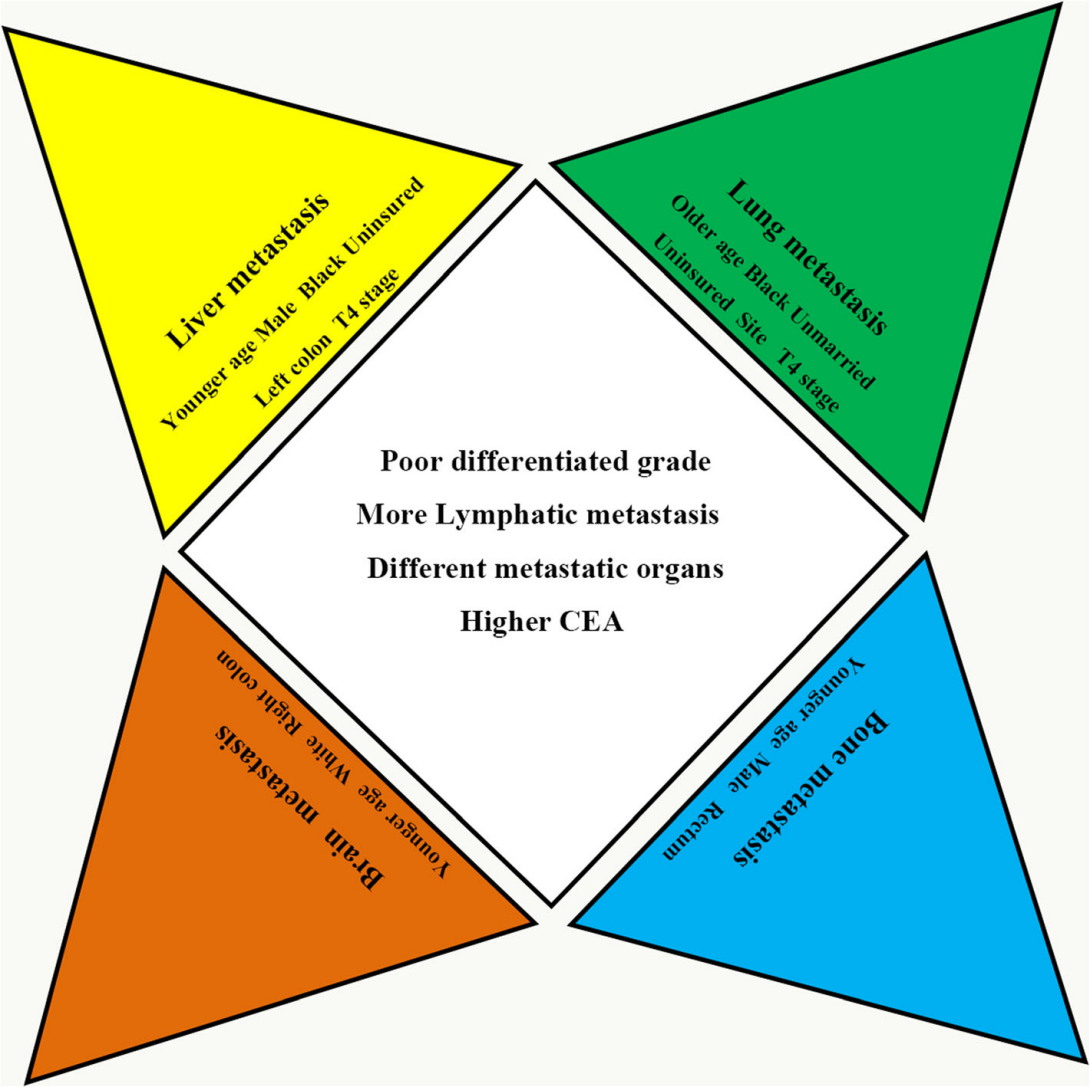
Homogeneous and heterogeneous associated factors of different types of metastases in patients with colorectal cancer. Factors of poor differentiation grade, more lymphatic metastasis, and CEA positivity were homogeneity associated factors for the four types of distant metastases. The factors listed in the angle exhibit the specific factors associated with each type of metastasis

Age, sex, race, marital status, insurance status, left/right colon, histological grade, lymphatic metastasis, T stage, and carcinoembryonic antigen (CEA) correlated with the occurrence of distant metastases by univariate analysis. The multivariable logistic regression model results indicated that male sex, black race, uninsured status, left/right colon, poor histological grade, T stage, and higher CEA were all positively associated with developing metastases (see Table 2). CRC exhibited homogeneity and heterogeneity for the factors associated with metastases in various organs. The associated factors for different sites of metastases are presented in Tables S1, S2, S3, S4. Poor differentiation grade, more lymphatic metastasis, higher CEA, and different metastatic organs were all positively associated with distant metastatic CRC. Younger age, male sex, black race, uninsured status, left/right colon, and T4/T1 stage were more positively associated with liver metastases. Older age, black race, uninsured status, site, and T4/T1 stage were more positively associated with lung metastases. Younger age, male sex, and rectum/right colon were more positively associated with bone metastases, and younger age, white race, and right colon were more positively associated with brain metastases (Fig. 3).

**Table 2:** Univariate and multivariate logistic regression for analysing the demographic and related clinical characteristics for developing distant metastases in patients diagnosed with colorectal cancer (diagnosed 2010–2016)

Subject characteristics	Univariate		Multivariate	
	OR (95% CI)	*P* value	OR (95% CI)	*P* value
Age(years)				
≤ 50	1 (reference)	1.00	1 (reference)	1.00
51–60	0.95 (0.92–0.99)	0.012	0.94 (0.90–0.98)	0.007
61–70	0.92 (0.88–0.95)	< 0.001	0.93 (0.89–0.97)	0.001
71–80	0.81 (0.78–0.85)	< 0.001	0.86 (0.82–0.90)	< 0.001
81–90	0.80 (0.77–0.83)	< 0.001	0.76 (0.72–0.80)	< 0.001
≥ 91	0.93 (0.86–1.01)	0.067	0.63 (0.58–0.70)	< 0.001
Sex				
Female	1 (reference)	1.00	1 (reference)	1.00
Male	1.16 (1.14–1.19)	< 0.001	1.21 (1.17–1.24)	< 0.001
Race				
White	1 (reference)	1.00	1 (reference)	1.00
Black	1.33 (1.29–1.38)	< 0.001	1.10 (1.06–1.14)	< 0.001
Others	0.96 (0.92–0.99)	0.040	0.82 (0.78–0.86)	< 0.001
Unknown	0.24 (0.19–0.29)	< 0.001	0.18 (0.15–0.23)	< 0.001
Marital status				
Unmarried	1 (reference)	1.00	1 (reference)	1.00
Married	0.84 (0.82–0.86)	< 0.001	0.98 (0.95–1.01)	0.151
Unknown	0.69 (0.65–0.72)	< 0.001	0.74 (0.69–0.78)	< 0.001
Insurance status				
Insured	1 (reference)	1.00	1 (reference)	1.00
Any Medic aid	1.45 (1.40–1.49)	< 0.001	1.16 (1.12–1.21)	< 0.001
Uninsured	1.78 (1.68–1.88)	< 0.001	1.32 (1.23–1.41)	< 0.001
Unknown	NA	NA	NA	NA
Site				
Right colon	1 (reference)	1.00	1 (reference)	1.00
Left colon	1.28 (1.25–1.32)	< 0.001	1.18 (1.14–1.22)	< 0.001
Rectum	0.99 (0.96–1.02)	0.628	0.84 (0.81–0.87)	< 0.001
Unknown	5.50 (5.21–5.80)	< 0.001	2.03 (1.90–2.17)	< 0.001
Histological grade				
Grade I	1 (reference)	1.00	1 (reference)	1.00
Grade II	2.19 (2.07–2.31)	< 0.001	1.81 (1.70–1.92)	< 0.001
Grade III	3.54 (3.33–3.77)	< 0.001	2.10 (1.96–2.25)	< 0.001
Grade IV	3.23 (2.96–3.52)	< 0.001	1.92 (1.75–2.12)	< 0.001
Unknown	7.13 (6.72–7.56)	< 0.001	3.24 (3.04–3.47)	< 0.001
Lymphatic metastasis				
N0	1 (reference)	1.00	1 (reference)	1.00
N1	2.69 (2.61–2.77)	< 0.001	0.29 (0.26–0.31)	< 0.001
N2	3.64 (3.52–3.76)	< 0.001	0.63 (0.60–0.66)	< 0.001
Unknown	10.50 (10.05–10.96)	< 0.001	1.10 (1.05–1.16)	< 0.001
T stage				
T1	1 (reference)	1.00	1 (reference)	1.00
T2	0.35 (0.32–0.38)	< 0.001	3.15 (3.04–3.26)	< 0.001
T3	1.50 (1.44–1.56)	< 0.001	4.22 (4.04–4.40)	< 0.001
T4	3.62 (3.46–3.78)	< 0.001	3.54 (3.36–3.73)	< 0.001
Unknown	8.11 (7.77–8.46)	< 0.001	3.15 (3.04–3.26)	< 0.001
CEA				
Negative	1 (reference)	1.00	1 (reference)	1.00
Positive	9.16 (8.81–9.52)	< 0.001	6.63 (6.37–6.91)	< 0.001
Unknown	2.15 (2.07–2.24)	< 0.001	1.57 (1.51–1.64)	< 0.001

*CEA* carcinoembryonic antigen, *NA* not available

^a^Includes American Indian/Alaska Native and Asian or Pacific Islander

^b^Includes single, separated, widowed, and divorced

### Nomograms for predicting distant metastases in CRC patients

**Fig. 4:**
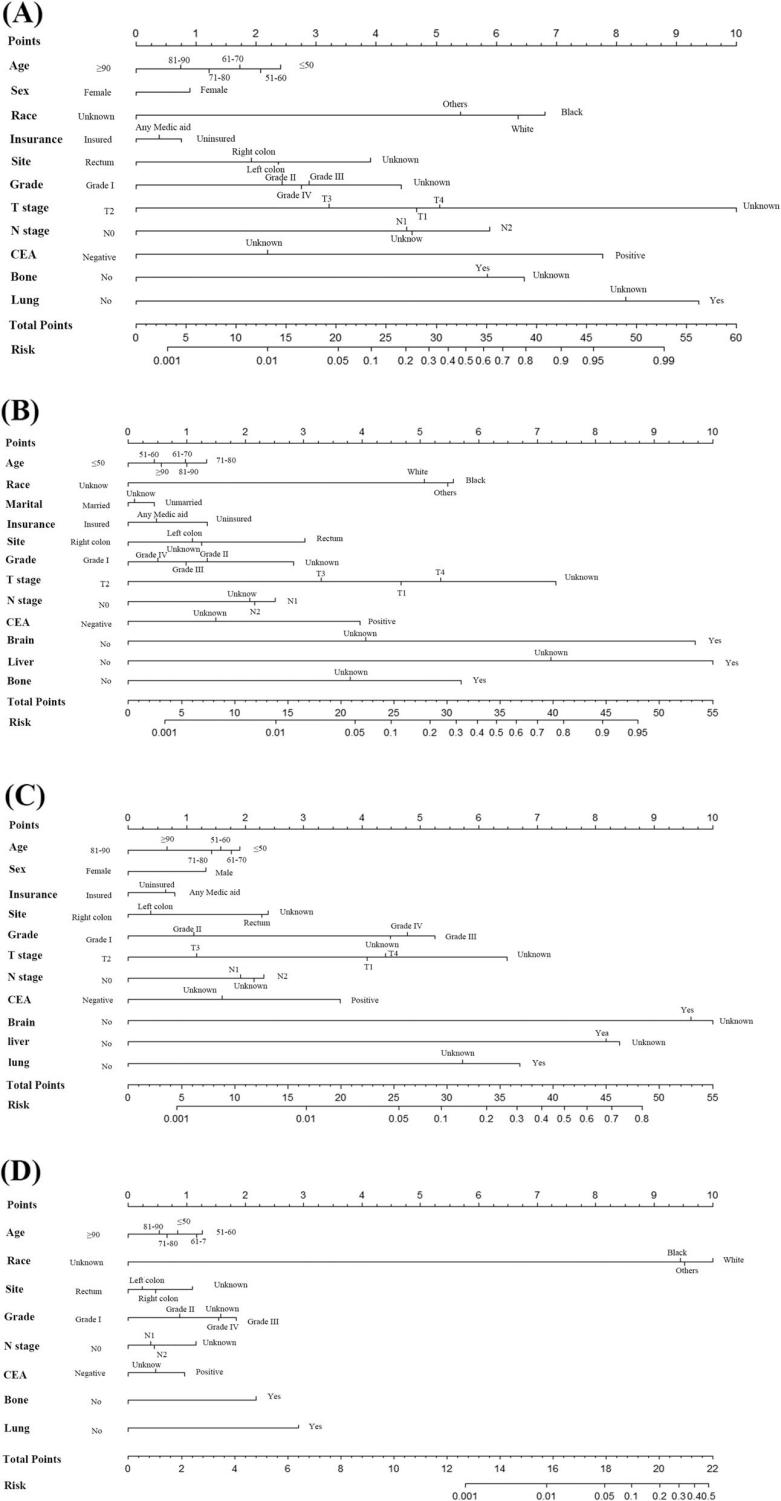
Nomogram for predicting (**a**) liver metastases, (**b**) lung metastases, (**c**) bone metastases, and (**d**) brain metastases

**Fig. 5:**
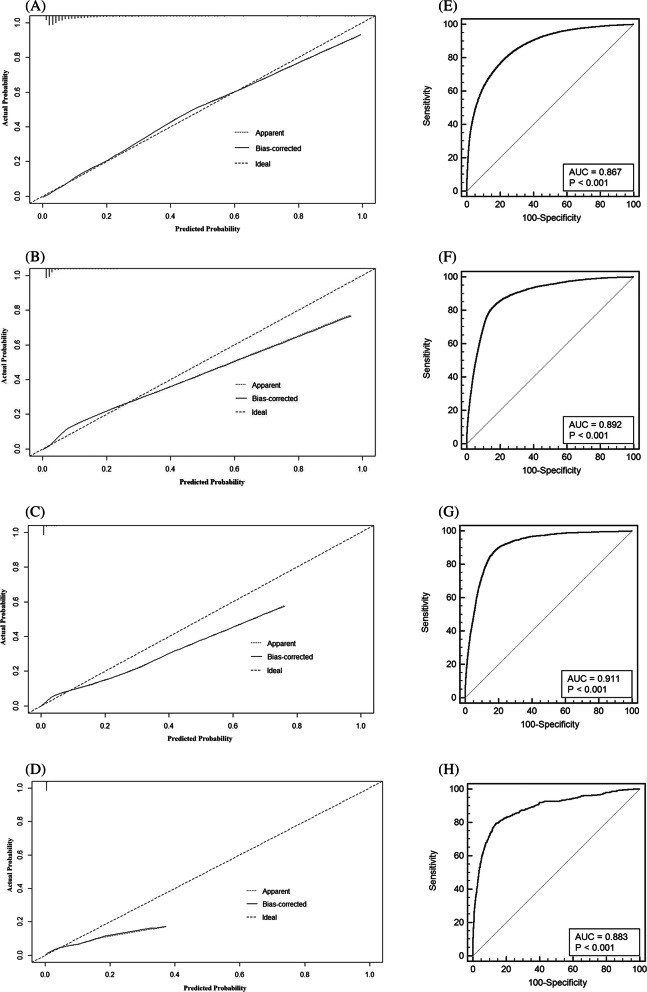
The calibration curve and ROC curve for assessing the calibration and discrimination of the nomogram in predicting liver metastases (**a**, **e**), lung metastases (**b**, **f**), bone metastases (**c**, **g**), and brain metastases (**d**, **h**)

Four nomograms were constructed with statistically significant variables to predict the probability of developing liver metastases, lung metastases, bone metastases, and brain metastases in CRC (Fig. 4a–d). The calibration curves showed good agreement between the predicted and observed probabilities (Fig. 5a–d). The ROC curve of the nomograms presented good discrimination for predicting these four metastases. The AUCs for these nomograms in liver metastases, lung metastases, bone metastases, and brain metastases were 86.7% (95% CI, 86.5–86.8%), 89.2% (95% CI, 89.0–89.3%), 91.1% (95% CI, 91.0–91.2%), and 88.3% (95% CI, 88.1–88.4%), respectively (Fig. 5e–h). The results of the ROC curve analysis revealed that the best thresholds were 0.114 with 83.1% sensitivity and 72.9% specificity, 0.036 with 84.0% sensitivity and 82.1% specificity, 0.010 with 88.4% sensitivity and 82.0% specificity, and 0.003 with 79.2% sensitivity and 86.3% specificity, respectively.

## Discussion

Previous studies reported the incidence of liver metastases, lung metastases, bone metastases, and brain metastases, which ranged from 14.5 to 26.5% [10–13], 2.4 to 6.9% [8, 14], 2.7 to 10% [4, 15, 16], and 0.23 to 3% [4, 6, 7, 15], respectively. Differences in the sample size of each study may have led to these inconsistent results for the same metastatic sites. To the best of our knowledge, the present study is the largest study on incidence, and we found that liver metastases were the most common metastatic pattern in CRC patients, followed by lung metastases and bone metastases. In contrast, metastases to the brain were relatively rare. This result is consistent with the above studies. The incidence of total distant metastases in our study was also similar to a previous study [17].

The present study also found that the incidence of distant metastases fluctuated with age. Our study proves that greater than 40% of patients developed distant metastases at the age of 51–70 years, and younger patients and older patients tended to have a lower prevalence. Screening programmes can identify patients at an early stage, and these programmes are cost effective [18]. Therefore, it is necessary to perform early screening for CRC patients [19, 20]. Our findings showed that males had a higher risk of developing distant metastases than females, and females had a higher incidence of right colon cancer. In contrast, males had a higher incidence of left colon cancer. Although some studies indicated sex and gender differences in colorectal cancer development [21, 22], the reasons for this difference are not clear. The biological and pathophysiological differences in CRC distant metastasis development between males and females must also be addressed in the future.

In addition to age and sex, the incidence of distant metastases was also different between CRC sites, which was seldom reported. The present study found that the highest incidence of distant metastases was observed for the sigmoid colon, followed by the rectum, cecum, ascending colon, rectosigmoid junction, transverse colon, descending colon, hepatic flexure, splenic flexure, and appendix. A German study found a similar anatomic site distribution, but that study investigated only colon cancers [23]. Regardless of colon cancer or metastatic CRC, these differences in incidence distribution may be due to molecular biological differences. For example, different anatomical sites exhibit different mutation rates in Ki-ras, p53, and epidermal growth factor receptor [24–26]. The present study also showed that colon cancer had a higher incidence of distant metastases than rectal cancer, which is partially consistent with a previous study [27]. Knowledge of these different behaviours based on primary sites may help guide targeted screening and introduce timely individualized interventions.

Previous studies showed that there were differences in the incidence of different organ metastases between different types of cancer and different histological types of the same cancer [28, 29]. The high incidence of distant metastases in CRC and different incidences in metastatic sites (liver, lung, bone, brain) may partially reflect the homogeneity and heterogeneity of distant metastases from CRC. The present study found that different metastatic sites showed homogeneity and heterogeneity in the factors associated with distant metastases from CRC. Four factors (poorly differentiated grade, more lymphatic metastasis, different metastatic organs, and higher CEA) were positively associated with the four types of metastases (liver, lung, bone, and brain). To our knowledge, the present study is the first study to describe these homogeneous factors for CRC distant metastases. These homogeneous associated factors may help with early detection in CRC patients and the development of individualized treatments to improve the prognosis.

However, the heterogeneous factors identified in our study are not entirely consistent with the results of previous studies. For example, we found that age, histological grade, and N stage were associated with brain metastases, which is opposite to previous findings [6]. However, we found that age, histological grade, serum levels of CEA, and the number of positive lymph nodes were associated with lung metastases from CRC, and male sex and rectal cancer were positively associated with bone metastases. These results are consistent with previous studies [5, 8].

The heterogeneities in the risk factors may partially be attributed to the different sample sizes because our study included over 200,000 CRC patients, which is greater than previous studies. More studies with larger sample sizes are needed. The biological and pathophysiological mechanisms behind the different risk factors specific to the site of metastasis of CRC are an important issue, but these issues are not clear. For example, male sex was a risk factor for bone metastases, which may be related to sex hormones and their receptors in the colon and lead to the differential development of colon cancer by sex [30, 31]. However, the detailed mechanism must be studied in the future.

The present study summarized the homogeneity and heterogeneity of the four types of metastatic CRC that were not comprehensively studied previously. The homogeneous and heterogeneous associated factors mentioned above may help in the surveillance of different types of distant metastases in CRC patients. To assist clinicians in identifying high-risk CRC patients, four predictive nomograms were constructed based on the factors associated with distant metastases. The results of internal validation revealed that the nomograms showed good prediction performance. Traditional early clinical metastatic screening and early diagnosis generally require extra techniques and equipment support, but predictive nomograms based on homogeneous and heterogeneous associated factors may be more cost effective. The nomograms provide a rapid metastatic screening tool. Previous studies have proven that nomograms provide considerable benefits to CRC patients, such as timely targeted therapy, improving the survival rate [32], and reducing the risk of emergency surgery [33]. Therefore, we recommend that CRC patients be screened using the predictive model first; then, the high-risk groups should be examined using PET scans or staging laparoscopies more frequently.

However, the present study has several limitations. A previous study found that never-smokers had a lower mortality risk than current smokers (HR 0.79, 95% CI, 0.64 to 0.99) among CRC patients [34], but smoking status was not included in our analysis. Some treatments, such as surgery, chemotherapy, and radiotherapy, were also not included. Our study investigated synchronous metastases. Surgery, radiotherapy, or chemotherapy will not affect synchronous metastases. Therefore, we did not include therapy in the development of distant metastases analysis. Other clinical factors, such as perforation and obstruction, were not studied because these factors were not available from the SEER database. However, these factors adversely impact outcomes and may affect the survival of CRC patients [35, 36]. The SEER database includes only the US population, and the results of this study may not be transferred to all other countries. The nomograms should be validated in other countries before they are used in specific countries.

## Conclusion

The present study demonstrated that 17.07% of CRC patients had distant metastases, and the incidences of liver metastases, lung metastases, bone metastases, and brain metastases were 15.34%, 5.22%, 1.26%, and 0.29%, respectively. The incidence of distant metastases was different by the age, gender, and various primary CRC sites. Poor differentiation grade, more lymphatic metastasis, different metastatic organs, and higher CEA were positively associated with these four types of distant metastases, and heterogeneous factors were also identified. Nomograms for predicting CRC patients with distant metastases were constructed. Although limited risk factors were included in this study, the established nomogram showed good prediction performance. These results may assist clinicians in identifying high-risk populations and providing individualized treatments.

## Supplementary Information

**Additional file 1: Table S1.** Univariate and multivariable logistic regression for analyzing the demographic and related clinical characteristics for developing liver metastasis in patients diagnosed with colorectal cancer (diagnosed 2010-2016). **Table S2.** Univariate and multivariable logistic regression for analyzing the demographic and related clinical characteristics for developing lung metastasis in patients diagnosed with colorectal cancer (diagnosed 2010-2016). **Table S3.** Univariate and multivariable logistic regression for analyzing the demographic and related clinical characteristics for developing bone metastasis in patients diagnosed with colorectal cancer (diagnosed 2010-2016). **Table S4.** Univariate and multivariable logistic regression for analyzing the demographic and related clinical characteristics for developing brain metastasis in patients diagnosed with colorectal cancer (diagnosed 2010-2016).

## Data Availability

The data that support the findings of this study are available from the corresponding author upon reasonable request.

## Abbreviations

CRC: Colorectal cancer
SEER: Surveillance, Epidemiology, and End Results
NCI: National Cancer Institute
SD: Standard deviation
ROC: Receiver operating characteristics
AUC: Area under the curve
SPSS: Statistical Package for the Social Sciences
CEA: Carcinoembryonic antigen
