# Supplementary material for: Nomogram based on homogeneous and heterogeneous associated factors for predicting distant metastases in patients with colorectal cancer
Source: World J Surg Oncol. 2021 Jan 27;19:30. doi: 10.1186/s12957-021-02140-6 (PMC7842036; doi:10.1186/s12957-021-02140-6)
Supplement: Supplementary file 1 — Additional file 1: Table S1. Univariate and multivariable logistic regression for analyzing the demographic and related clinical characteristics for developing liver metastasis in patients diagnosed with colorectal cancer (diagnosed 2010-2016). Table S2. Univariate and multivariable logistic regression for analyzing the demographic and related clinical characteristics for developing lung metastasis in patients diagnosed with colorectal cancer (diagnosed 2010-2016). Table S3. Univariate and multivariable logistic regression for analyzing the demographic and related clinical characteristics for developing bone metastasis in patients diagnosed with colorectal cancer (diagnosed 2010-2016). Table S4. Univariate and multivariable logistic regression for analyzing the demographic and related clinical characteristics for developing brain metastasis in patients diagnosed with colorectal cancer (diagnosed 2010-2016). [file 12957_2021_2140_MOESM1_ESM.doc]

**SUPPORTING INFORMATION**

**Table S1** Univariate and multivariable logistic regression for analyzing the demographic and related clinical characteristics for developing liver metastasis in patients diagnosed with colorectal cancer (diagnosed 2010-2016).

**Table S2** Univariate and multivariable logistic regression for analyzing the demographic and related clinical characteristics for developing lung metastasis in patients diagnosed with colorectal cancer (diagnosed 2010-2016).

**Table S3** Univariate and multivariable logistic regression for analyzing the demographic and related clinical characteristics for developing bone metastasis in patients diagnosed with colorectal cancer (diagnosed 2010-2016).

**Table** **S4** Univariate and multivariable logistic regression for analyzing the demographic and related clinical characteristics for developing brain metastasis in patients diagnosed with colorectal cancer (diagnosed 2010-2016).

**Table S1** Univariate and multivariable logistic regression for analyzing the demographic and related clinical characteristics for developing liver metastasis in patients diagnosed with colorectal cancer (diagnosed 2010-2016).

| Subject characteristics | Univariate | | | Multivariate | |
| --- | --- | --- | --- | --- | --- |
|  | OR (95%CI) | | P-value | OR (95%CI) | P-value |
| Age(years) | | | | | |
| ≤50 | 1(Reference) | | 1.00 | 1(Reference) | 1.00 |
| 51-60 | 1.06(0.99-1.12) | | 0.087 | 0.93(0.89-0.97) | ﹤0.001 |
| 61-70 | 0.99(0.93-1.06) | | 0.787 | 0.86(0.82-0.90) | ﹤0.001 |
| 71-80 | 0.87(0.82-0.93) | | ﹤0.001 | 0.76(0.73-0.80) | ﹤0.001 |
| 81-90 | 0.85(0.80-0.91) | | ﹤0.001 | 0.69(0.65-0.73) | ﹤0.001 |
| ≥91 | 0.97(0.88-1.07) | | 0.562 | 0.58(0.52-0.64) | ﹤0.001 |
| Sex | | | | | |
| Female | 1(Reference) | | 1.00 | 1(Reference) | 1.00 |
| Male | 1.18(1.15-1.21) | | ﹤0.001 | 1.23(1.19-1.27) | ﹤0.001 |
| Race | | | | | |
| White | 1(Reference) | | 1.00 | 1(Reference) | 1.00 |
| Black | 1.36(1.31-1.40) | | ﹤0.001 | 1.12(1.07-1.16) | ﹤0.001 |
| Others | 0.94(0.91-0.99) | | 0.07 | 0.80(0.76-0.84) | ﹤0.001 |
| Unknown | 0.24(0.19-0.30) | | ﹤0.001 | 0.24(0.19-0.30) | ﹤0.001 |
| Marital status | | | | | |
| Unmarried | 1(Reference) | | 1.00 | 1(Reference) | 1.00 |
| Married | 0.85(0.83-0.87) | | ﹤0.001 | 1.01(0.98-1.04) | 0.53 |
| Unknown | 0.68(0.64-0.72) | | ﹤0.001 | 0.76(0.71-0.81) | ﹤0.001 |
| Insurance status | | | | | |
| Insured | | 1(Reference) | 1.00 | 1(Reference) | 1.00 |
| Any Medic aid | | 1.42(1.37-1.47) | ﹤0.001 | 1.09(1.04-1.13) | ﹤0.001 |
| Uninsured | | 1.76(1.66-1.86) | ﹤0.001 | 1.19(1.11-1.28) | ﹤0.001 |
| Site | | | | | |
| Right colon | | 1(Reference) | 1.00 | 1(Reference) | 1.00 |
| Left colon | | 1.27(1.23-1.30) | ﹤0.001 | 1.11(1.08-1.15) | ﹤0.001 |
| Rectum | | 0.89(0.86-0.92) | ﹤0.001 | 0.64(0.62-0.67) | ﹤0.001 |
| Unknown | | 4.92(4.66-5.19) | ﹤0.001 | 1.58(1.47-1.70) | ﹤0.001 |
| Histological grade | | | | | |
| Grade I | | 1(Reference) | 1.00 | 1(Reference) | 1.00 |
| Grade II | | 2.22(2.09-2.36) | ﹤0.001 | 1.75(1.63-1.87) | ﹤0.001 |
| Grade III | | 3.51(3.29—3.74) | ﹤0.001 | 1.94(1.80-2.09) | ﹤0.001 |
| Grade IV | | 3.27(2.99-3.58) | ﹤0.001 | 1.88(1.69-2.09) | ﹤0.001 |
| Unknown | | 6.94(6.52-7.39) | ﹤0.001 | 2.80(2.60-3.01) | ﹤0.001 |
| Lymphatic metastasis | | | | | |
| N0 | | 1(Reference) | 1.00 | 1(Reference) | 1.00 |
| N1 | | 2.69(2.61-2.77) | ﹤0.001 | 2.82(2.71-2.93) | ﹤0.001 |
| N2 | | 3.68(3.55-3.81) | ﹤0.001 | 3.90(3.72-4.08) | ﹤0.001 |
| Unknown | | 9.90(9.48-10.35) | ﹤0.001 | 2.66(2.50-2.82) | ﹤0.001 |
| T stage | | | | | |
| T1 | | 1(Reference) | 1.00 | 1(Reference) | 1.00 |
| T2 | | 0.35(0.32-0.38) | ﹤0.001 | 0.34(0.31-0.37) | ﹤0.001 |
| T3 | | 1.50(1.43-1.56) | ﹤0.001 | 0.71(0.67-0.75) | ﹤0.001 |
| T4 | | 3.54(3.38-3.71) | ﹤0.001 | 1.10(1.03-1.15) | 0.004 |
| Unknown | | 7.78(7.44-8.14) | ﹤0.001 | 3.39(3.20-3.58) | ﹤0.001 |
| CEA | | | | | |
| Negative | | 1(Reference) | 1.00 | 1(Reference) | 1.00 |
| Positive | | 9.76(9.36-10.17) | ﹤0.001 | 6.06(5.79-6.34) | ﹤0.001 |
| Unknown | | 2.30(2.21-2.41) | ﹤0.001 | 1.67(1.59-1.75) | ﹤0.001 |
| Lung Metastasis | | | | | |
| None | | 1(Reference) | 1.00 | 1(Reference) | 1.00 |
| Yes | | 19.89(19.02-20.81) | ﹤0.001 | 8.79(8.34-9.26) | ﹤0.001 |
| Unknown | | 26.59(23.32-30.31) | ﹤0.001 | 6.01(5.07-7.14) | ﹤0.001 |
| Bone Metastasis | | | | | |
| None | | 1(Reference) | 1.00 | 1(Reference) | 1.00 |
| Yes | | 15.92(14.57-17.39) | ﹤0.001 | 3.82(3.43-4.26) | ﹤0.001 |
| Unknown | | 26.42(22.86-30.53) | ﹤0.001 | 2.02(1.54-2.65) | ﹤0.001 |
| Brain Metastasis | | | | | |
| None | | 1(Reference) | 1.00 | 1(Reference) | 1.00 |
| Yes | | 6.99(5.93-8.25) | ﹤0.001 | 0.85(0.68-1.05) | 0.132 |
| Unknown | | 27.19(23.58-31.36) | ﹤0.001 | 3.04(2.34-3.95) | ﹤0.001 |

Abbreviations: CEA=carcinoembryonic antigen;

a Includes American Indian/Alaska Native and Asian or Pacific Islander;

b Includes single, separated, widowed, and divorced.

**Table S2** Univariate and multivariable logistic regression for analyzing the demographic and related clinical characteristics for developing lung metastasis in patients diagnosed with colorectal cancer (diagnosed 2010-2016).

| Subject characteristics | Univariate | | Multivariate | | |
| --- | --- | --- | --- | --- | --- |
|  | OR (95%CI) | P-value | OR (95%CI) | | P-value |
| Age(years) | | | | | |
| ≤50 | 1(Reference) | 1.00 | 1(Reference) | | 1.00 |
| 51-60 | 1.09(1.02-1.16) | 0.013 | 1.11(1.03-1.19) | | 0.007 |
| 61-70 | 1.09(1.03-1.17) | 0.005 | 1.24(1.16-1.34) | | ﹤0.001 |
| 71-80 | 1.02(0.96-1.09) | 0.525 | 1.34(1.24-1.45) | | ﹤0.001 |
| 81-90 | 0.96(0.89-1.04) | 0.321 | 1.24(1.14-1.36) | | ﹤0.001 |
| ≥91 | 1.09(0.95-1.25) | 0.213 | 1.13(0.96-1.32) | | 0.132 |
| Sex | | | | | |
| Female | 1(Reference) | 1.00 | 1(Reference) | | 1.00 |
| Male | 1.10(1.05-1.14) | ﹤0.001 | 0.93(0.84-1.03) | | 0.142 |
| Race | | | | | |
| White | 1(Reference) | 1.00 | 1(Reference) | | 1.00 |
| Black | 1.36(1.29-1.44) | ﹤0.001 | 1.12(1.05-1.19) | | 0.001 |
| Others | 1.10(1.03-1.17) | 0.006 | 1.09(1.01-1.18) | | 0.022 |
| Unknown | 0.21(0.14-0.32) | ﹤0.001 | 0.33(0.21-0.50) | | ﹤0.001 |
| Marital status | | | | | |
| Unmarried | 1(Reference) | 1.00 | 1(Reference) | | 1.00 |
| Married | 0.77(0.74-0.81) | ﹤0.001 | 0.91(0.87-0.95) | | ﹤0.001 |
| Unknown | 0.73(0.66-0.79) | ﹤0.001 | 0.93(0.84-1.03) | | 0.139 |
| Insurance status | | | | | |
| Insured | 1(Reference) | 1.00 | 1(Reference) | | 1.00 |
| Any Medic aid | 1.48(1.40-1.56) | ﹤0.001 | 1.11(1.05-1.18) | | 0.001 |
| Uninsured | 1.88(1.72-2.05) | ﹤0.001 | 1.35(1.22-1.50) | | ﹤0.001 |
| Site | | | | | |
| Right colon | 1(Reference) | 1.00 | 1(Reference) | | 1.00 |
| Left colon | 1.41(1.35-1.49) | ﹤0.001 | 1.23(1.21-1.35) | | ﹤0.001 |
| Rectum | 1.64(1.56-1.73) | ﹤0.001 | 1.96(1.84-2.08) | | ﹤0.001 |
| Unknown | 5.22(4.83-5.63) | ﹤0.001 | 1.32(1.20-1.45) | | ﹤0.001 |
| Histological grade | | | | | |
| Grade I | 1(Reference) | 1.00 | 1(Reference) | | 1.00 |
| Grade II | 1.94(1.76-2.14) | ﹤0.001 | 1.35(1.21-1.50) | | ﹤0.001 |
| Grade III | 2.54(2.27-2.83) | ﹤0.001 | 1.25(1.10-1.40) | | ﹤0.001 |
| Grade IV | 2.01(1.70-2.36) | ﹤0.001 | 1.12(0.93-1.34) | | 0.229 |
| Unknown | 6.65(6.01-7.35) | ﹤0.001 | 1.87(1.67-2.09) | | ﹤0.001 |
| Lymphatic metastasis | | | | | |
| N0 | 1(Reference) | 1.00 | 1(Reference) | | 1.00 |
| N1 | 2.37(2.26-2.48) | ﹤0.001 | 1.74(1.64-1.85) | | ﹤0.001 |
| N2 | 2.18(2.05-2.31) | ﹤0.001 | 1.61(1.49-1.74) | | ﹤0.001 |
| Unknown | 7.93(7.47-8.42) | ﹤0.001 | 1.58(1.47-1.70) | | ﹤0.001 |
| T stage | | | | | |
| T1 | 1(Reference) | 1.00 | 1(Reference) | | 1.00 |
| T2 | 0.25(0.21-0.30) | ﹤0.001 | 0.36(0.30-0.42) | | ﹤0.001 |
| T3 | 1.04(0.96-1.11) | 0.343 | 0.74(0.68-0.80) | | ﹤0.001 |
| T4 | 2.51(2.34-2.71) | ﹤0.001 | 1.16(1.07-1.27) | | 0.001 |
| Unknown | 6.25(5.84-6.68) | ﹤0.001 | 1.80(1.66-1.94) | | ﹤0.001 |
| CEA | | | | | |
| Negative | 1(Reference) | 1.00 | | 1(Reference) | 1.00 |
| Positive | 8.41(7.84-9.03) | ﹤0.001 | | 2.40(2.22-2.59) | ﹤0.001 |
| Unknown | 2.42(2.24-2.60) | ﹤0.001 | | 1.39(1.28-1.51) | ﹤0.001 |
| Liver Metastasis | | | | | |
| None | 1(Reference) | 1.00 | | 1(Reference) | 1.00 |
| Yes | 19.89(19.02-20.81) | ﹤0.001 | | 9.13(8.66-9.62) | ﹤0.001 |
| Unknown | 22.59(18.35-27.8) | ﹤0.001 | | 4.95(3.83-6.40) | ﹤0.001 |
| Bone Metastasis | | | | | |
| None | 1(Reference) | 1.00 | 1(Reference) | | 1.00 |
| Yes | 16.62(15.31-18.05) | ﹤0.001 | 3.53(3.21-3.88) | | ﹤0.001 |
| Unknown | 19.24(16.62-22.28) | ﹤0.001 | 2.32(1.80-2.99) | | ﹤0.001 |
| Brain Metastasis | | | | | |
| None | 1(Reference) | 1.00 | 1(Reference) | | 1.00 |
| Yes | 21.71(18.38-25.63) | ﹤0.001 | 8.53(6.94-10.48) | | ﹤0.001 |
| Unknown | 19.05(16.57-21.90) | ﹤0.001 | 2.45(1.94-3.10) | | ﹤0.001 |

Abbreviations: CEA=carcinoembryonic antigen;

a Includes American Indian/Alaska Native and Asian or Pacific Islander;

b Includes single, separated, widowed, and divorced.

**Table S3** Univariate and multivariable logistic regression for analyzing the demographic and related clinical characteristics for developing bone metastasis in patients diagnosed with colorectal cancer (diagnosed 2010-2016).

| Subject characteristics | Univariate | | Multivariate | |
| --- | --- | --- | --- | --- |
|  | OR (95%CI) | P-value | OR (95%CI) | P-value |
| Age(years) | | | | |
| ≤50 | 1(Reference) | 1.00 | 1(Reference) | 1.00 |
| 51-60 | 1.00(0.89-1.14) | 0.909 | 0.94(0.82-1.07) | 0.347 |
| 61-70 | 0.99(0.88-1.12) | 0.869 | 0.98(0.86-1.11) | 0.663 |
| 71-80 | 0.86(0.75-0.98) | 0.019 | 0.91(0.79-1.05) | 0.199 |
| 81-90 | 0.68(0.58-0.79) | ﹤0.001 | 0.69(0.58-0.82) | ﹤0.001 |
| ≥91 | 0.91(0.69-1.20) | 0.505 | 0.79(0.58-1.08) | 0.119 |
| Sex | | | | |
| Female | 1(Reference) | 1.00 | 1(Reference) | 1.00 |
| Male | 1.39(1.28-1.50) | ﹤0.001 | 1.28(1.17-1.40) | ﹤0.001 |
| Race | | | | |
| White | 1(Reference) | 1.00 | 1(Reference) | 1.00 |
| Black | 1.30(1.17-1.45) | ﹤0.001 | 1.04(0.93-1.17) | 0.466 |
| Others | 0.99(0.87-1.14) | 0.90 | 0.99(0.85-1.14) | 0.851 |
| Unknown | 0.23(0.11-0.52) | ﹤0.001 | 0.38(0.17-0.86) | 0.021 |
| Marital status | | | | |
| Unmarried | 1(Reference) | 1.00 | 1(Reference) | 1.00 |
| Married | 0.85(0.79-0.92) | ﹤0.001 | 1.02(0.93-1.11) | 0.714 |
| Unknown | 0.72(0.60-0.87) | ﹤0.001 | 0.88(0.73-1.07) | 0.211 |
| Insurance status | | | | |
| Insured | 1(Reference) | 1.00 | 1(Reference) | 1.00 |
| Any Medic aid | 1.57(1.42-1.74) | ﹤0.001 | 1.16(1.03-1.30) | 0.009 |
| Uninsured | 1.78(1.49-2.12) | ﹤0.001 | 1.11(0.92-1.35) | 0.209 |
| Site | | | | |
| Right colon | 1(Reference) | 1.00 | 1(Reference) | 1.00 |
| Left colon | 1.28(1.15-1.41) | ﹤0.001 | 1.08(0.97-1.21) | 0.155 |
| Rectum | 1.61(1.45-1.79) | ﹤0.001 | 1.55(1.38-1.74) | ﹤0.001 |
| Unknown | 6.67(5.86-7.60) | ﹤0.001 | 1.57(1.35-1.83) | ﹤0.001 |
| Histological grade | | | | |
| Grade I | 1(Reference) | 1.00 | 1(Reference) | 1.00 |
| Grade II | 1.64(1.32-2.04) | ﹤0.001 | 1.23(0.98-1.55) | 0.075 |
| Grade III | 4.49(3.58-5.64) | ﹤0.001 | 2.68(2.11-3.40) | ﹤0.001 |
| Grade IV | 3.50(2.58-4.75) | ﹤0.001 | 2.46(1.78-3.39) | ﹤0.001 |
| Unknown | 8.68(6.98-10.79) | ﹤0.001 | 2.33(1.85-2.93) | ﹤0.001 |
| Lymphatic metastasis | | | | |
| N0 | 1(Reference) | 1.00 | 1(Reference) | 1.00 |
| N1 | 2.10(1.90-2.31) | ﹤0.001 | 1.44(1.29-1.61) | ﹤0.001 |
| N2 | 2.07(1.83-2.32) | ﹤0.001 | 1.55(1.34-1.80) | ﹤0.001 |
| Unknown | 8.42(7.56-9.39) | ﹤0.001 | 1.51(1.33-1.71) | ﹤0.001 |
| T stage | | | | |
| T1 | 1(Reference) | 1.00 | 1(Reference) | 1.00 |
| T2 | 0.29(0.21-0.39) | ﹤0.001 | 0.46(0.34-0.63) | ﹤0.001 |
| T3 | 0.72(0.62-0.83) | ﹤0.001 | 0.57(0.49-0.67) | ﹤0.001 |
| T4 | 2.30(1.99-2.66) | ﹤0.001 | 1.06(0.90-1.24) | 0.514 |
| Unknown | 6.23(5.46-7.09) | ﹤0.001 | 1.57(1.36-1.82) | ﹤0.001 |
| CEA | | | | |
| Negative | 1(Reference) | 1.00 | 1(Reference) | 1.00 |
| Positive | 7.82(6.76-9.04) | ﹤0.001 | 2.00(1.71-2.33) | ﹤0.001 |
| Unknown | 2.60(2.23-3.02) | ﹤0.001 | 1.37(1.17-1.61) | ﹤0.001 |
| Liver Metastasis | | | | |
| None | 1(Reference) | 1.00 | 1(Reference) | 1.00 |
| Yes | 15.92(14.57-17.39) | ﹤0.001 | 4.65(4.17-5.18) | ﹤0.001 |
| Unknown | 32.31(23.55-44.32) | ﹤0.001 | 4.90(3.31-7.26) | ﹤0.001 |
| Lung Metastasis | | | | |
| None | 1(Reference) | 1.00 | 1(Reference) | 1.00 |
| Yes | 16.62(15.31-18.05) | ﹤0.001 | 3.54(3.22-3.90) | ﹤0.001 |
| Unknown | 20.59(16.57-25.60) | ﹤0.001 | 2.97(2.29-3.85) | ﹤0.001 |
| Brain Metastasis | | | | |
| None | 1(Reference) | 1.00 | 1(Reference) | 1.00 |
| Yes | 26.8722.01-32.80) | ﹤0.001 | 6.16(4.91-7.73) | ﹤0.001 |
| Unknown | 42.08(33.55-52.77) | ﹤0.001 | 6.65(5.07-8.71) | ﹤0.001 |

Abbreviations: CEA=carcinoembryonic antigen;

a Includes American Indian/Alaska Native and Asian or Pacific Islander;

b Includes single, separated, widowed, and divorced.

**Table S4** Univariate and multivariable logistic regression for analyzing the demographic and related clinical characteristics for developing brain metastasis in patients diagnosed with colorectal cancer (diagnosed 2010-2016).

| Subject characteristics | Univariate | | Multivariate | |
| --- | --- | --- | --- | --- |
|  | OR (95%CI) | P-value | OR (95%CI) | P-value |
| Age(years) | | | | |
| ≤50 | 1(Reference) | 1.00 | 1(Reference) | 1.00 |
| 51-60 | 1.37(1.05-1.80) | 0.021 | 1.32(1.00-1.74) | 0.049 |
| 61-70 | 1.36(1.05-1.77) | 0.022 | 1.25(0.96-1.65) | 0.104 |
| 71-80 | 0.98(0.73-1.31) | 0.089 | 0.89(0.65-1.21) | 0.448 |
| 81-90 | 0.86(0.61-1.20) | 0.36 | 0.72(0.51-1.03) | 0.07 |
| ≥91 | 0.67(0.32-1.38) | 0.27 | 0.41(0.19-0.86) | 0.019 |
| Sex | | | | |
| Female | 1(Reference) | 1.00 | 1(Reference) | 1.00 |
| Male | 1.02(0.87-1.20) | 0.837 | 0.91(0.77-1.08) | 0.27 |
| Race | | | | |
| White | 1(Reference) | 1.00 | 1(Reference) | 1.00 |
| Black | 0.88(0.68-1.13) | 0.322 | 0.64(0.49-0.83) | 0.001 |
| Others | 0.75(0.55-1.02) | 0.066 | 0.73(0.53-1.00) | 0.049 |
| Unknown | 0.00 | 0.986 | 0.00 | 0.986 |
| Marital status | | | | |
| Unmarried | 1(Reference) | 1.00 | 1(Reference) | 1.00 |
| Married | 0.84(0.71-0.99) | 0.041 | 0.97(0.81-1.16) | 0.746 |
| Unknown | 0.69(0.47-1.00) | 0.054 | 0.82(0.55-1.22) | 0.329 |
| Insurance status | | | | |
| Insured | 1(Reference) | 1.00 | 1(Reference) | 1.00 |
| Any Medic aid | 1.40(1.13-1.74) | 0.002 | 1.07(0.86-1.35) | 0.54 |
| Uninsured | 1.70(1.18-2.44) | 0.004 | 1.10(0.75-1.62) | 0.621 |
| Site | | | | |
| Right colon | 1(Reference) | 1.00 | 1(Reference) | 1.00 |
| Left colon | 0.98(0.80-1.20) | 0.848 | 0.83(0.67-1.04) | 0.082 |
| Rectum | 0.96(0.77-1.21) | 0.739 | 0.73(0.57-0.94) | 0.012 |
| Unknown | 6.12(4.78-7.83) | ﹤0.001 | 1.47(1.08-1.99) | 0.007 |
| Histological grade | | | | |
| Grade I | 1(Reference) | 1.00 | 1(Reference) | 1.00 |
| Grade II | 2.27(1.37-3.78) | ﹤0.001 | 1.84(1.10-3.08) | 0.021 |
| Grade III | 5.85(3.48-9.87) | ﹤0.001 | 3.58(2.10-6.12) | ﹤0.001 |
| Grade IV | 4.35(2.22-8.52) | ﹤0.001 | 2.93(1.47-5.84) | 0.002 |
| Unknown | 9.89(5.96-16.43) | ﹤0.001 | 2.70(1.59-4.56) | ﹤0.001 |
| Lymphatic metastasis | | | | |
| N0 | 1(Reference) | 1.00 | 1(Reference) | 1.00 |
| N1 | 2.02(1.64-2.49) | ﹤0.001 | 1.44(1.15-1.81) | 0.001 |
| N2 | 2.21(1.72-2.83) | ﹤0.001 | 1.71(1.28-2.29) | ﹤0.001 |
| Unknown | 9.12(7.33-11.34) | ﹤0.001 | 1.80(1.40-2.32) | ﹤0.001 |
| T stage | | | | |
| T1 | 1(Reference) | 1.00 | 1(Reference) | 1.00 |
| T2 | 0.58(0.35-0.96) | 0.035 | 0.81(0.48-1.36) | 0.429 |
| T3 | 1.03(0.76-1.41) | 0.854 | 0.83(0.59-1.16) | 0.273 |
| T4 | 2.18(1.57-3.01) | ﹤0.001 | 0.96(0.67-1.37) | 0.819 |
| Unknown | 6.62(4.98-8.81) | ﹤0.001 | 1.78(1.30-2.44) | ﹤0.001 |
| CEA |  |  |  |  |
| Negative | 1(Reference) | 1.00 | 1(Reference) | 1.00 |
| Positive | 5.06(3.88-6.60) | ﹤0.001 | 1.73(1.30-2.31) | ﹤0.001 |
| Unknown | 2.02(1.53-2.66) | ﹤0.001 | 1.26(0.94-1.68) | 0.119 |
| Liver Metastasis | | | | |
| None | 1(Reference) | 1.00 | 1(Reference) | 1.00 |
| Yes | 6.99(5.93-8.25) | ﹤0.001 | 1.13(0.92-1.40) | 0.248 |
| Unknown | 27.46(16.41-45.94) | ﹤0.001 | 2.60(1.36-4.97) | 0.004 |
| Lung Metastasis | | | | |
| None | 1(Reference) | 1.00 | 1(Reference) | 1.00 |
| Yes | 21.71(18.38-25.63) | ﹤0.001 | 7.67(6.21-9.48) | ﹤0.001 |
| Unknown | 20.67(13.18-32.42) | ﹤0.001 | 3.66(2.11-6.34) | ﹤0.001 |
| Bone Metastasis | | | | |
| None | 1(Reference) | 1.00 | 1(Reference) | 1.00 |
| Yes | 26.87(22.01-32.80) | ﹤0.001 | 5.04(4.03-6.29) | ﹤0.001 |
| Unknown | 59.97(40.28-89.28) | ﹤0.001 | 10.91(6.75-17.65) | ﹤0.001 |

Abbreviations: CEA=carcinoembryonic antigen;

a Includes American Indian/Alaska Native and Asian or Pacific Islander;

b Includes single, separated, widowed, and divorced.
